# AGING IN COMMUNITY: A LEGAL MAPPING ANALYSIS OF MASSACHUSETTS MUNICIPAL ACCESSORY DWELLING UNIT ZONING BYLAWS

**DOI:** 10.1093/geroni/igz038.939

**Published:** 2019-11-08

**Authors:** Travis M Gagen

**Affiliations:** Assumption College, Worcester, Massachusetts, United States

## Abstract

Accessory-dwelling units (ADUs) are one alternative housing arrangement that enable older adults to remain in the home despite functional decline. Functional decline increases with age making older adults more susceptible to loosing independent housing. Involuntary relocation to institutional care can result in a decline of functional health, reduced life satisfaction, impairment of psychological well-being and increased mortality rate. The majority of older Americans (93%) wish to remain in their home for as long as possible. ADUs function to maintain, stimulate and support an older adult as a means to prevent relocation to an institution. The modified environment coupled with adaptable features maintains and supports activities of daily living (ADL) within a familiar place. Under Massachusetts law MGL c. 40A, the state gives authority to cities and towns to adopt ordinances and bylaws to regulate the use of land, buildings and structures. Restrictive zoning laws limit the ability to construct health-promoting built-environments to age-in-community. All 351 Massachusetts municipalities Accessory Dwelling Unit (ADU) zoning bylaws were coded using the ADU Friendliness Score. Once scored, the 351 municipalities were placed into four categories based off their ADU score; the four categories are poor (0-24), fair (25-49), good (50-74), and excellent (75-100). Eighty-nine municipalities (25%) are in the poor category; thirty municipalities (8.5%) are in the fair category; one hundred and eighty-five municipalities (53%) are in the good category; forty-seven municipalities (13.5%) are in the excellent category. These findings contributed to a model ADU bylaw specific for aging Americans for municipalities to adopt.

